# Vinexin family (SORBS) proteins regulate mechanotransduction in mesenchymal stem cells

**DOI:** 10.1038/s41598-018-29700-3

**Published:** 2018-08-01

**Authors:** Mito Kuroda, Kazumitsu Ueda, Noriyuki Kioka

**Affiliations:** 1 Division of Applied Life Sciences, Graduate School of Agriculture, Kyoto University, Sakyo, Kyoto 606-8502 Japan; 2 Institute for Integrated Cell-Material Sciences (iCeMS), Kyoto University, Sakyo, Kyoto 606-8507 Japan

## Abstract

The stiffness of extracellular matrix (ECM) directs the differentiation of mesenchymal stem cells (MSCs) through the transcriptional co-activators Yes-associated protein (YAP) and transcriptional coactivator with a PDZ-binding motif (TAZ). Although a recent study revealed the involvement of vinexin α and CAP (c-Cbl-associated proteins), two of vinexin (SORBS) family proteins that bind to vinculin, in mechanosensing, it is still unclear whether these proteins regulate mechanotransduction and differentiation of MSCs. In the present study, we show that both vinexin α and CAP are necessary for the association of vinculin with the cytoskeleton and the promotion of YAP/TAZ nuclear localization in MSCs grown on rigid substrates. Furthermore, CAP is involved in the MSC differentiation in a stiffness-dependent manner, whereas vinexin depletion suppresses adipocyte differentiation independently of YAP/TAZ. These observations reveal a critical role of vinexin α and CAP in mechanotransduction and MSC differentiation.

## Introduction

Extracellular matrix (ECM) stiffness has emerged as a critical regulator of cellular responses, such as cell migration^1–4^, proliferation^5^, and differentiation^6^. For instance, cells migrate more rapidly on rigid substrates as a short-term response. Mesenchymal stem cells (MSCs) preferentially differentiate into adipocytes on soft substrates, whereas they differentiate into osteoblasts on rigid substrates as a long-term response. Mechanisms by which cells sense ECM stiffness (mechanosensing) and transduce the information to downstream signaling pathways (mechanotransduction) have been receiving increasing attention^7^.

Cell-ECM adhesion sites, called focal adhesions (FAs), mechanically link the ECM to the actin cytoskeleton and play critical roles in mechanosensing and mechanotransduction. FAs contain ECM receptor proteins, integrins, and cytosolic adaptor proteins, including talin and vinculin. Force-induced conformational changes in FA proteins are thought to be key steps in the mechanism by which physical cues are transduced into biochemical signals^8^. For example, substrate domains of p130CAS (Crk-associated substrate) are extended in response to cell stretching, leading to CAS phosphorylation by Src family kinases^9^. Talin rod domains adjacent to the N-terminal head domain are unfolded by a tensile force, enabling the vinculin-binding site (VBS) of talin to bind to vinculin^10^.

Vinculin is another major sensor for ECM stiffness and consists of an N-terminal head region and a C-terminal tail region, connected by a proline-rich linker region. Intramolecular interactions between the head and the tail regions (i.e., closed form of vinculin) suppress interactions with binding partners, including F-actin, resulting in a low affinity for F-actin, while disruption of the interaction leads to conformational changes of vinculin into a structure with a high affinity for F-actin (i.e., open form of vinculin)^11,12^. Culturing on rigid substrates as well as myosin activity induce the conformational change of vinculin into the open form and the immobilization of vinculin at FAs^4,13–15^. The F-actin-binding ability of vinculin is involved in this process^16^. Furthermore, the vinculin conformational change induced by ECM stiffness contributes to the differentiation of MSCs in a manner dependent on ECM stiffness^17^.

The ECM stiffness-dependent regulation of vinculin requires the binding of its proline-rich linker region to other FA proteins, vinexin α (also known as SORBS3) or c-Cbl-associated protein (CAP) (also known as SORBS1 or ponsin) in mouse embryonic fibroblasts (MEFs)^4,18^. Furthermore, vinexin is required for ECM stiffness-dependent cell migration^4^. Vinexin and CAP, together with Arg-binding protein 2 (ArgBP2) (also known as SORBS2)^19,20^, constitute a SORBS protein family. These proteins share the same domain structures, containing a sorbin homology (SoHo) domain and three Src homology 3 (SH3) domains (Fig. S1A). SORBS family proteins exhibit some functional redundancy, including sharing binding partners and their similar roles in ECM stiffness-dependent regulation of vinculin^18,21–27^. However, the downstream signals and phenotypes of knockout (KO) mice differ from each other: Vinexin KO mice show delayed wound healing and increased cardiac hypertrophy^20,28^. CAP plays a role in PI3K-independent insulin signaling^25,29^, and CAP KO mice show improved insulin resistance under high fat feeding^30^. ArgBP2 is involved in generating intracellular tension^18,31^, and ArgBP2 KO mice show impaired long-term memory^32^. However, it remains unclear whether SORBS proteins regulate MSC differentiation in an ECM stiffness-dependent manner.

The transcriptional coactivators, Yes-associated protein (YAP)/ transcriptional coactivator with a PDZ-binding motif (TAZ), have been intensely investigated as mechanotransducers that regulate both stem cell differentiation and cancer progression^33,34^. When grown on soft substrates YAP/TAZ are sequestered in cytoplasm, whereas they localize in nucleus when grown on rigid substrates. This regulation involves FA, actin cytoskeleton and nucleoskeleton^33,35,36^. Depletion of vinculin, talin, or actin-binding FA proteins decrease YAP/TAZ nuclear localization on rigid ECM^17,35,37^. However, upstream regulators of YAP/TAZ are incompletely understood.

In the present study, we show that vinexin and CAP are involved in the regulation of the ECM stiffness-dependent nuclear localization of YAP/TAZ in MSCs. In addition, CAP regulates the differentiation of MSCs into adipocytes dependent on ECM stiffness.

## Results

### Vinexin and CAP contribute to the cytoskeletal association of vinculin in MSCs

We first examined the expression of vinexin family proteins in ST2 cells, a mouse mesenchymal stem cell line (Fig. S1B). The expressions of vinexin α, its transcriptional variant, vinexin β, and CAP were detected using western blotting, while ArgBP2 expression was not detected (data not shown). Thus, we focused on vinexin and CAP in this study. To investigate the roles of vinexin and CAP in the MSC differentiation, vinexin and CAP expression were stably knocked down using lentiviruses carrying shRNAs. Expression of vinexin α was reduced to less than 1% of its original value in two vinexin-depleted cell lines (#1 and #2) (Fig. S1B). CAP is known to have several splice variants^38^, and the most prominent signal was detected at approximately 130 kDa in ST2 cells (Fig. S1B). The expression of CAP variants was reduced to 6.4% (#1) and 13% (#2) in CAP-depleted cells (Fig. S1B). Immunostaining analysis demonstrated a decrease in the amounts of vinexin or CAP localized in both the cytosol and FAs in each knockdown cells (Fig. S1C,D).

**Figure 1:**
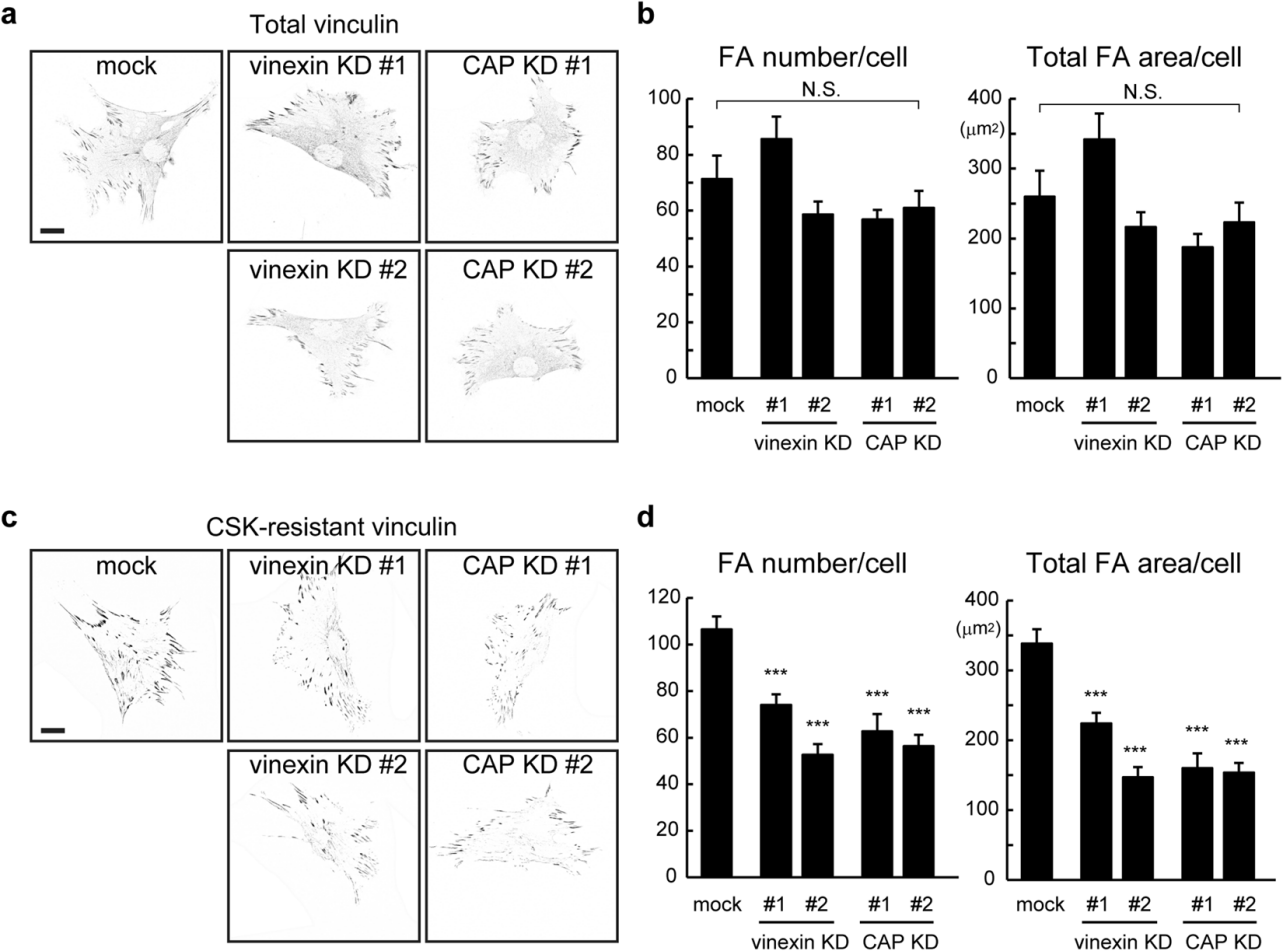
Vinexin and CAP were required for the cytoskeletal association of vinculin in MSCs. (**a**–**d**) Control (mock), vinexin-depleted cells (vinexin KD) and CAP-depleted cells (CAP KD) on glass substrates coated with 10 μg/ml type-I collagen at a density of 1.0 × 10^4^ cells/well in 12-well plates were fixed without (**a**,**b**) or with (**c**,**d**) CSK treatment. Cells were immunostained with anti-vinculin antibody. Images were obtained using confocal microscopy, and the gray images were inverted to increase their visibility (**a**,**c**). The number of FAs and total FA area in each cell were quantified using ImageJ (**b**,**d**). Fifteen cells from two separate experiments were analyzed. The values represent the mean ± s.e.m. Statistical significance was determined by one-way ANOVA with Tukey’s test (**b**,**d**). ***p < 0.001. Scale bars: 20 μm.

Vinexin α and CAP contribute to the CSK (cytoskeleton stabilization buffer)-resistance of vinculin, which represents the fraction of vinculin that tightly binds to the cytoskeleton (i.e., open form of vinculin), in MEFs grown on rigid substrates^18^. Thus, we first asked whether vinexin or CAP is required for the vinculin localization at FAs and the cytoskeletal association of vinculin in ST2 cells on rigid (glass) substrates. Vinculin localization was examined using normal immunostaining (Fig. 1a). Quantified data showed that the numbers and total area of FAs per cell in vinexin- and CAP-depleted cells were comparable with those of control cells, indicating that vinexin and CAP are dispensable for vinculin localization at FAs in ST2 cells (Fig. 1b). On the other hand, the numbers and area of FAs containing CSK-resistant vinculin decreased significantly in vinexin- and CAP-depleted cells (Fig. 1c,d). In addition, the plasma membrane was stained using Cell Mask Orange to quantify the cell-spreading area and aspect ratio (Fig. S2A). No significant differences in cell area and aspect ratio were observed between control and vinexin- or CAP-depleted cells (Fig. S2B). Taken together, these results suggest that vinexin and CAP are required for the cytoskeletal association of vinculin in MSCs on rigid substrates.

### Vinexin and CAP promote the nuclear localization of YAP/TAZ on rigid ECM

**Figure 2:**
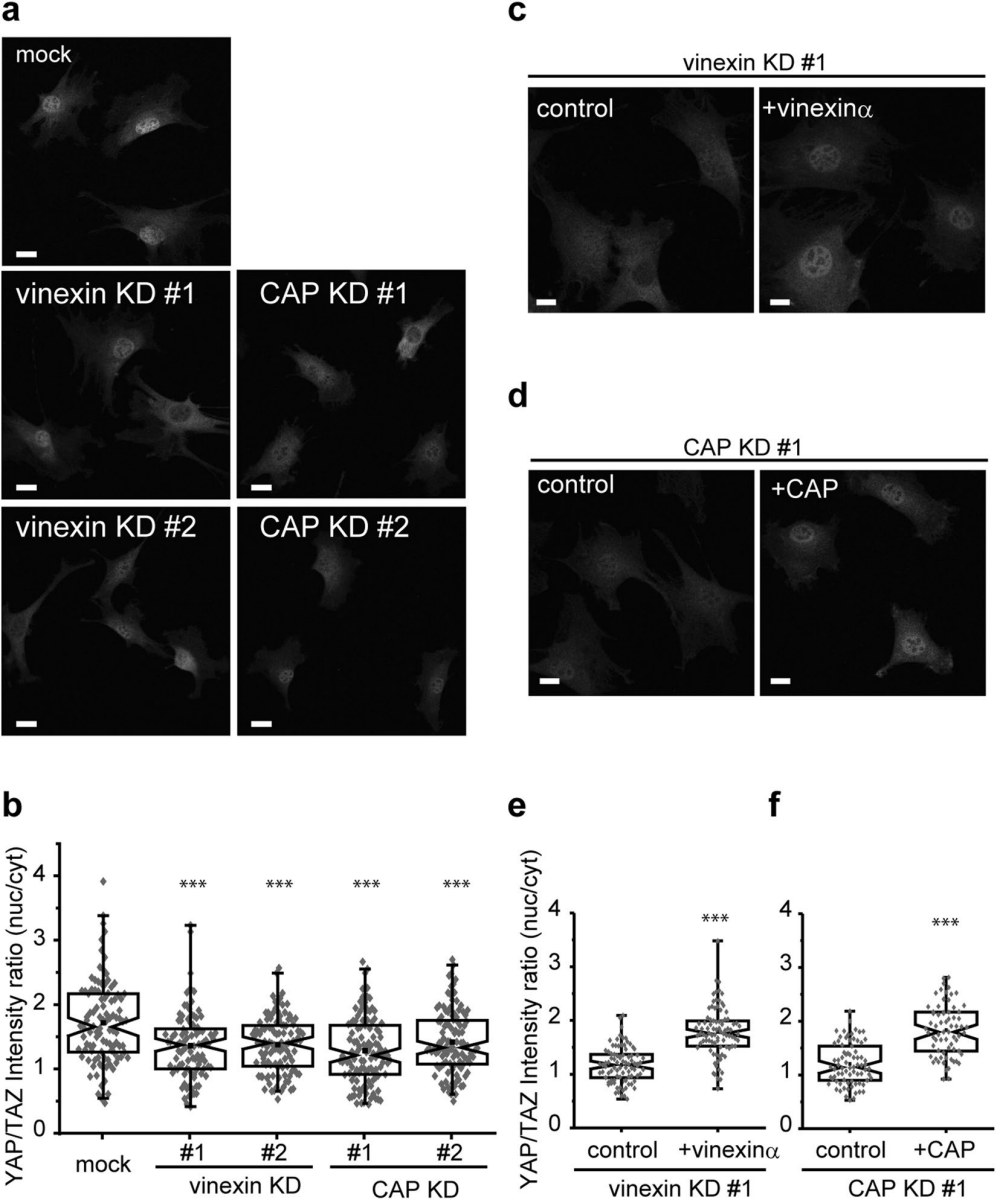
Vinexin and CAP promoted the nuclear localization of YAP/TAZ. (**a**,**b**) Cells were seeded on glass substrates coated with 10 μg/ml collagen I in 12-well plates at a density of 2.0 × 10^4^ cells/well and fixed 24 hours after seeding. Cells were immunostained with anti-YAP/TAZ antibody and nuclei were visualized with Hoechst 33342 staining. (**b**) The ratio (nuc/cyt) of YAP/TAZ staining intensity was quantified. Mock: n = 102, vinexin KD#1: n = 97, vinexin KD #2: n = 109, CAP KD#1: n = 117, CAP KD#2: n = 112. (**c**–**f**) Control, and vinexin α- or CAP- re-expressing KD cells were seeded on glass substrates and immunostained with anti YAP-TAZ antibody. (**e**,**f**) The intensity ratio (nuc/cyt) of YAP/TAZ from two separate experiments was quantified. Vinexin KD#1 + control: n = 87, + vinexinα: n = 89. CAP KD#1 + control: n = 77 + CAP: n = 69. Each experiment was performed twice. For each box plot, the box boundaries represent the 25th–75th percentiles, and the whiskers represent the 1st and 99th percentile. Notches on the box represent the confidential interval about the median value. The center dot represents the mean. Statistical significance was determined by Kruskal–Wallis ANOVA with Mann–Whitney’s U-test (**b**,**e**,**f**). ***p < 0.001. Scale bars: 20 μm.

Vinculin promotes the nuclear localization of the transcriptional co-activators, YAP/TAZ, in an ECM stiffness-dependent manner, whereas a mutation in the proline-rich linker region, wherein both vinexin and CAP bind^22^, attenuates this effect^17^. Therefore, we investigated the effects of vinexin and CAP on the nuclear localization of YAP/TAZ. Vinexin- and CAP-depleted cells were seeded onto rigid (glass) substrates and immunostained using an anti-YAP/TAZ antibody (Fig. 2a). Both vinexin and CAP depletion decreased YAP/TAZ nuclear localization. Quantitative analyses indicate that the ratio of YAP/TAZ nuclear to cytosolic intensity were decreased in the vinexin- and CAP-depleted cells when compared with that in control cells (Fig. 2b). No significant differences were observed in the nuclear to cytosolic intensity ratio between vinexin- and CAP-depleted cells. To confirm these data, vinexin-depleted cells re-expressing vinexin α (referred to as vinexin α re-expressing cells) and CAP-depleted cells re-expressing CAP (referred to as CAP re-expressing cells) were established (Fig. S1E,F). Re-expression of vinexin α and CAP rescued the decrease in the nuclear localization of YAP/TAZ (Fig. 2c–f). These results indicate that vinexin and CAP promote the nuclear localization of YAP/TAZ in cells grown on rigid substrates.

**Figure 3:**
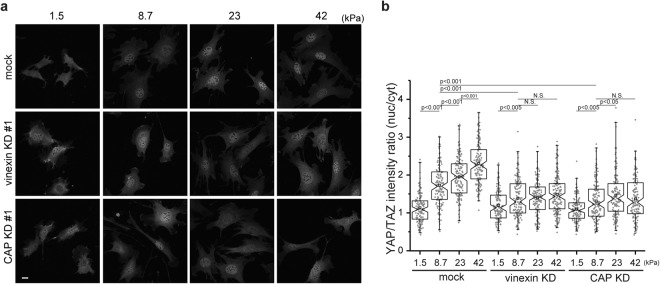
Vinexin and CAP promoted the nuclear localization of YAP/TAZ in an ECM stiffness-dependent manner. (**a**,**b**) Control cells and vinexin- or CAP-depleted cells (6.0 × 10^4^ cells/35 mm dish) on collagen coated PAA gels with different levels of stiffness (1.5 kPa, 8.7 kPa, 23 kPa, 42 kPa) were immunostained with anti-YAP/TAZ antibody. (**b**) The quantified intensity ratio are shown. Mock: n = 114, 130, 128, 132, vinexin KD#1: n = 132, 132, 140, 141, CAP KD#1: n = 121, 135, 128, 131. The experiment was repeated twice. Statistical significance was determined by Kruskal–Wallis’s ANOVA with Mann–Whitney’s U-test. Scale bars: 20 μm.

To explore the effect of vinexin and CAP depletion on ECM stiffness-regulated YAP/TAZ nuclear localization, vinexin- and CAP-depleted cells were seeded onto polyacrylamide (PAA) gels with different levels of stiffness, ranging from 1.5 kPa to 42 kPa (Fig. 3). Consistent with a previous report^17^, substrates with moderate rigidity (8.7 kPa) increased the nuclear to cytosolic intensity ratio to 1.72 ± 0.05 compared with on soft substrates (1.13 ± 0.04, 1.5 kPa) in control cells (Fig. 3b). Stiffer substrates (23 kPa and 42 kPa) further increased the intensity ratio to 1.95 ± 0.05 and 2.31 ± 0.05, respectively. In contrast, the increases in the intensity ratios in vinexin- and CAP-depleted cells on substrates with a stiffness of 8.7 kPa were only moderate, 1.39 ± 0.04 and 1.30 ± 0.04, respectively. In addition, no further increase was observed in vinexin-depleted cells on gels with a stiffness above 8.7 kPa. A similar tendency was observed in CAP-depleted cells. Taken together, these observations suggest that both vinexin and CAP are required to promote the YAP/TAZ nuclear localization in cells grown on rigid substrates.

### CAP inhibits cell differentiation into adipocytes, whereas vinexin exerts the opposite effect

On rigid substrates, vinculin inhibits the adipocyte differentiation and promotes the osteoblast differentiation by promoting nuclear localization of YAP/TAZ^17^. Thus, we investigated the roles of vinexin and CAP in the regulation of MSC differentiation. We first investigated the temporal expression patterns of vinexin and CAP using western blotting in wild-type (WT) ST2 cells during differentiation into either adipocytes or osteoblasts (Fig. S2C). Expression of vinexin α increased, prior to the induction of differentiation (day 0), possibly due to cell density. Vinexin α expression was maintained during the progression of adipocyte differentiation but decreased during the progression of osteoblast differentiation. Expression of vinexin β remained constant throughout differentiation into both adipocytes and osteoblasts. In contrast, the expression of CAP decreased prior to the induction of differentiation (day 0) and upregulated during adipogenesis and osteogenesis. These results suggest a role of SORBS proteins during the differentiation of MSCs.

**Figure 4:**
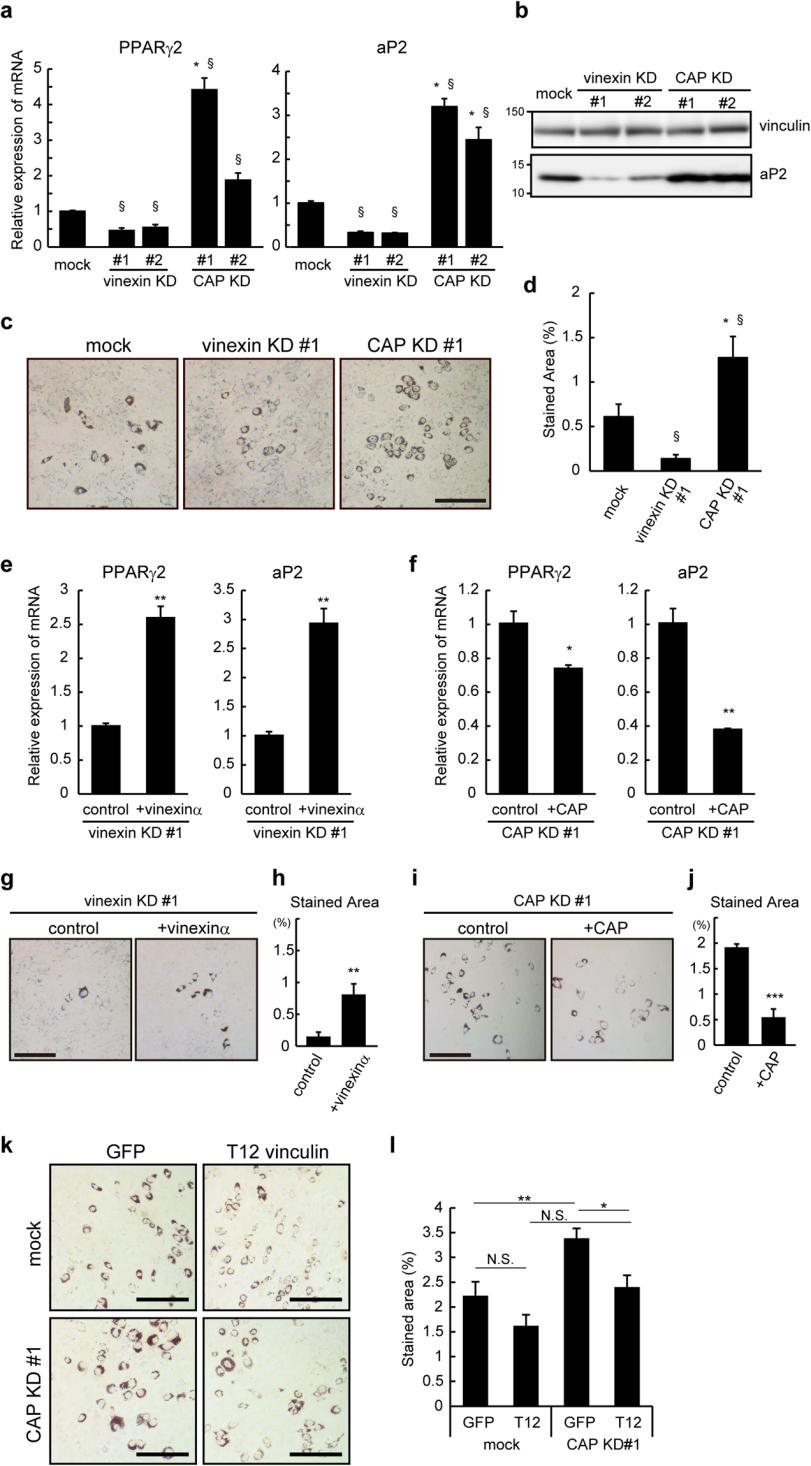
CAP suppressed, but vinexin promoted, adipocyte differentiation. Vinexin- and CAP-depleted (**a**–**d**) cells, as well as vinexin α re-expressing cells (**e**,**g**,**h**) and CAP re-expressing cells (**f**,**i**,**j**) were seeded on plastic dishes at a density of 1.2 × 10^5^ cells/35 mm dish and induced to differentiate into adipocyte. RNAs were extracted 6 days after MDI treatment, and the mRNAs expression of PPARγ2 and aP2 was quantified by qRT-PCR (**a**,**e**,**f**). The expression levels relative to those in control cells are shown. (**b**) Cell were lysed and equal amounts of cell lysates were analyzed by western blotting using the anti-aP2 and anti-vinculin (loading control) antibodies. Blots were cropped from full-size images (see Supplemental Information). (**c**,**d**,**g**–**j**) Cells were stained with Oil Red O on day 6 after MDI treatment and representative images are shown (**c**,**g**,**i**). (**d**,**h**,**j**) Nine images were obtained from three independent experiments, and the percentage of area stained were quantified using ImageJ. (**k**,**l**) Control and CAP depleted cells at the density of 1.0 × 10^5^ cells/ 6 well plates were infected with lentiviruses expressing GFP-T12 vinculin. Two days after the infection, cells were induced to differentiate into adipocytes. Differentiated adipocytes were stained with Oil Red O on day 6. (l) Twelve images were obtained from two independent experiments, and the percentage of the stained area was quantified using ImageJ. Scale bars: 200 μm. The values represent the mean ± s.e.m. Statistical significance was determined by one-way ANOVA with Tukey’s test or Student’s t-test (**e**,**f**,**h**,**j**). *p < 0.05, **p < 0.01. ***p < 0.001 (by Tukey’s test). ^§^p < 0.001 (compared to mock by Student’s t-test).

To elucidate the roles of vinexin and CAP in the differentiation of MSCs, we tested the differentiation of vinexin- and CAP-depleted cells into adipocytes on rigid (plastic) substrates. qRT-PCR analysis showed that depletion of CAP increased the expression of adipogenic markers, PPARγ2 (4.4 ± 0.01-fold (#1) and 1.9 ± 0.01-fold (#2)) and aP2 (3.2 ± 0.06-fold (#1) and 2.4 ± 0.12-fold (#2)) (Fig. 4a). Unexpectedly, depletion of vinexin decreased the expression of adipogenic markers PPARγ2 (0.5 ± 0.17-fold (#1) and 0.6 ± 0.14-fold (#2)) and aP2 (0.3 ± 0.10-fold (#1) and 0.3 ± 0.04-fold (#2)). Western blotting analysis also showed that CAP depletion increased expression of aP2 protein whereas vinexin-depletion decreased it (Fig. 4b). Furthermore, Oil Red O staining analysis showed an increase in lipid accumulation by CAP depletion but a decrease by vinexin depletion (Fig. 4c,d). Re-expression of vinexin α or CAP rescued the effects of depletion, as revealed by qRT-PCR analysis (Fig. 4e,f) and Oil Red O staining (Fig. 4g–j). Similar results were obtained in vinexin-depleted murine C3H10T1/2 cell lines (data not shown), supporting that vinexin plays a role in promoting adipogenesis. Collectively, these results demonstrate that CAP inhibits the differentiation of MSCs into adipocytes on rigid substrates, whereas vinexin exerts the opposite effect.

To address whether cytoskeletal association of vinculin at FAs induced by CAP is involved in the regulation of MSC differentiation, vinculin T12, a semi-open and constitutively CSK-resistant vinculin mutant^12,16,39^, was introduced into CAP depleted cells and the adipocyte differentiation was examined (Fig. 4k,l). As shown in Fig. 4l, expression of GFP-T12 vinculin in CAP depleted cells suppressed the accumulation of lipid droplets to the levels comparable to control cells. In contrast, GFP-T12 suppressed the accumulation only slightly in control cells. These results suggest that CAP induces the cytoskeletal association of vinculin at FAs to suppress the adipocyte differentiation on rigid substrates.

### CAP promotes, but vinexin suppresses, differentiation into osteoblasts

**Figure 5:**
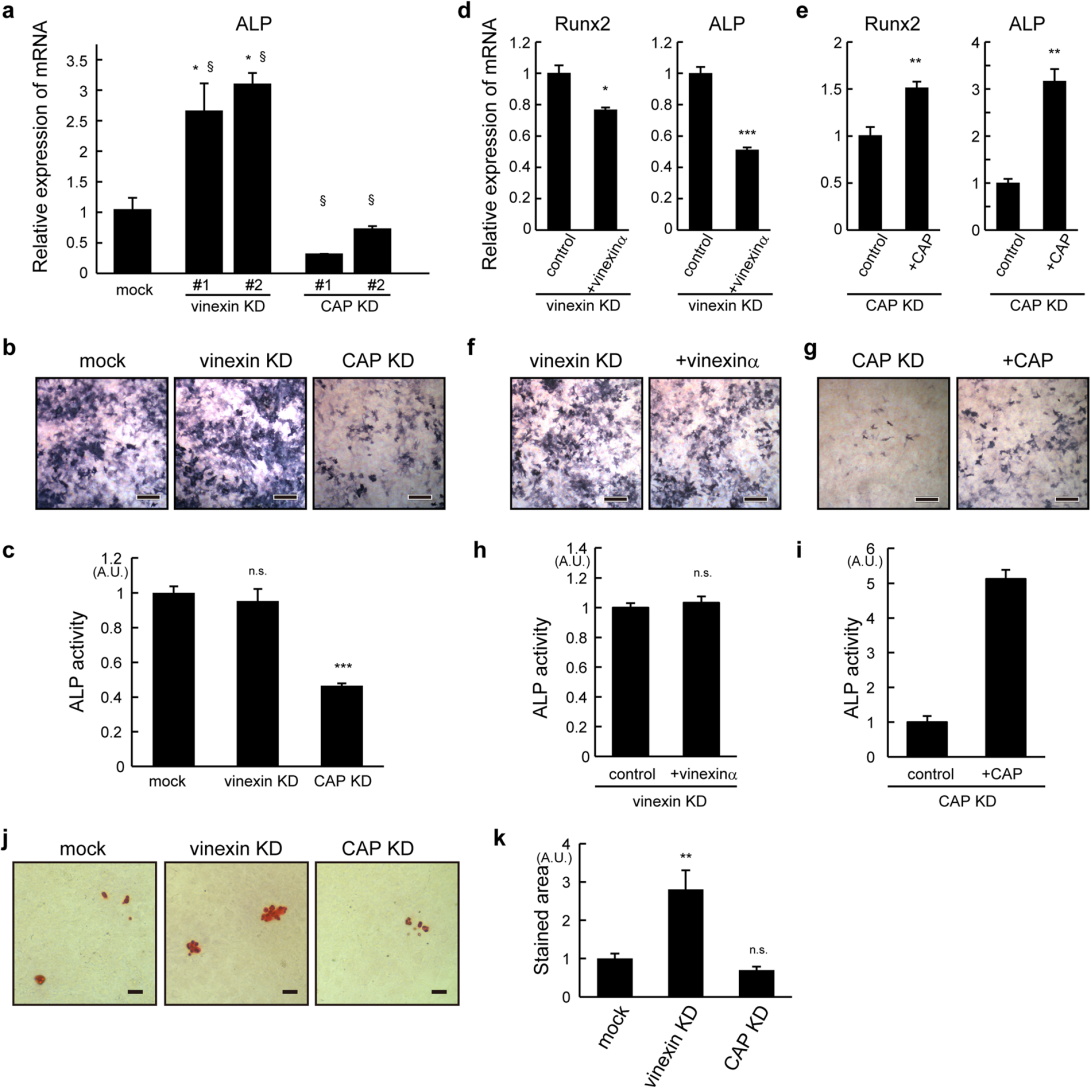
CAP promoted osteoblast differentiation and vinexin inhibited calcification. Vinexin- or CAP-depleted (**a**,**b**,**c**,**j**,**k**) cells and vinexin α re-expressing cells (**d**,**f**,**h**) or CAP re-expressing cells seeded on plastic dishes were induced to differentiate into osteoblasts by incubating them with αMEM medium. (**a**,**d**,**e**) On day 6, RNAs were extracted and the expression of the ALP mRNA was quantified by qRT-PCR. The relative expression compared to control cells is shown from triplicate experiment. (**b**,**c**,**f**–**i**) On day 4, ALP activity was visualized and six images from each condition were obtained from two independent experiments. Representative images are shown. Scale bars: 400 μm. (**c**,**h**,**i**) Total intensity was quantified using ImageJ. (**j**,**k**) On day 6, cells were stained with Alizarin Red S and six images were obtained from two independent experiments. Representative images are shown. Scale bars: 200 μm. (**k**) The relative stained area was quantified using ImageJ. Values represent the mean ± s.e.m. Statistical significance was determined by one-way ANOVA with Tukey’s test (**a**,**c**,**k**) and Student’s t-test (**d**,**e**,**h**,**i**). ***p < 0.001 (by Tukey’s test). ^§^p < 0.01 (compared to mock using Student’s t-test).

We next investigated the effects of vinexin and CAP on osteoblast differentiation. Vinexin- or CAP-depleted cells were seeded onto rigid (plastic) substrates and differentiation into osteoblasts was induced. The mRNA expression of alkaline phosphatase (ALP), a marker for osteoblast differentiation, was down-regulated in CAP-depleted cells, whereas it was upregulated in vinexin-depleted cells (Fig. 5a). The osteoblastic phenotype was further analyzed using ALP-activity staining (Fig. 5b,c). Quantified data showed that CAP depletion significantly decreased the ALP-activity staining compared to control cells (Fig. 5c). On the other hand, vinexin depletion did not affect the staining. Re-expression of CAP rescued the decrease in ALP mRNA expression and activity staining (Fig. 5e,g,i). Re-expression of vinexin α rescued the mRNA expression but, again, did not affect ALP activity (Fig. 5d,f,h). The reason why vinexin depletion has different effects on ALP mRNA and activity staining is not clear. Post-transcriptional regulation could explain the difference, since ALP activity can be regulated post-transcriptionally^40^. Next, Alizarin Red S staining was performed to examine the effect on calcification. Vinexin depletion increased the staining compared to that in control cells (Fig. 5j,k), whereas CAP depletion slightly decreased the staining. Thus, these results suggest that CAP promotes differentiation into osteoblasts, whereas vinexin suppresses the differentiation.

### CAP regulates ECM stiffness-dependent differentiation into adipocytes

**Figure 6:**
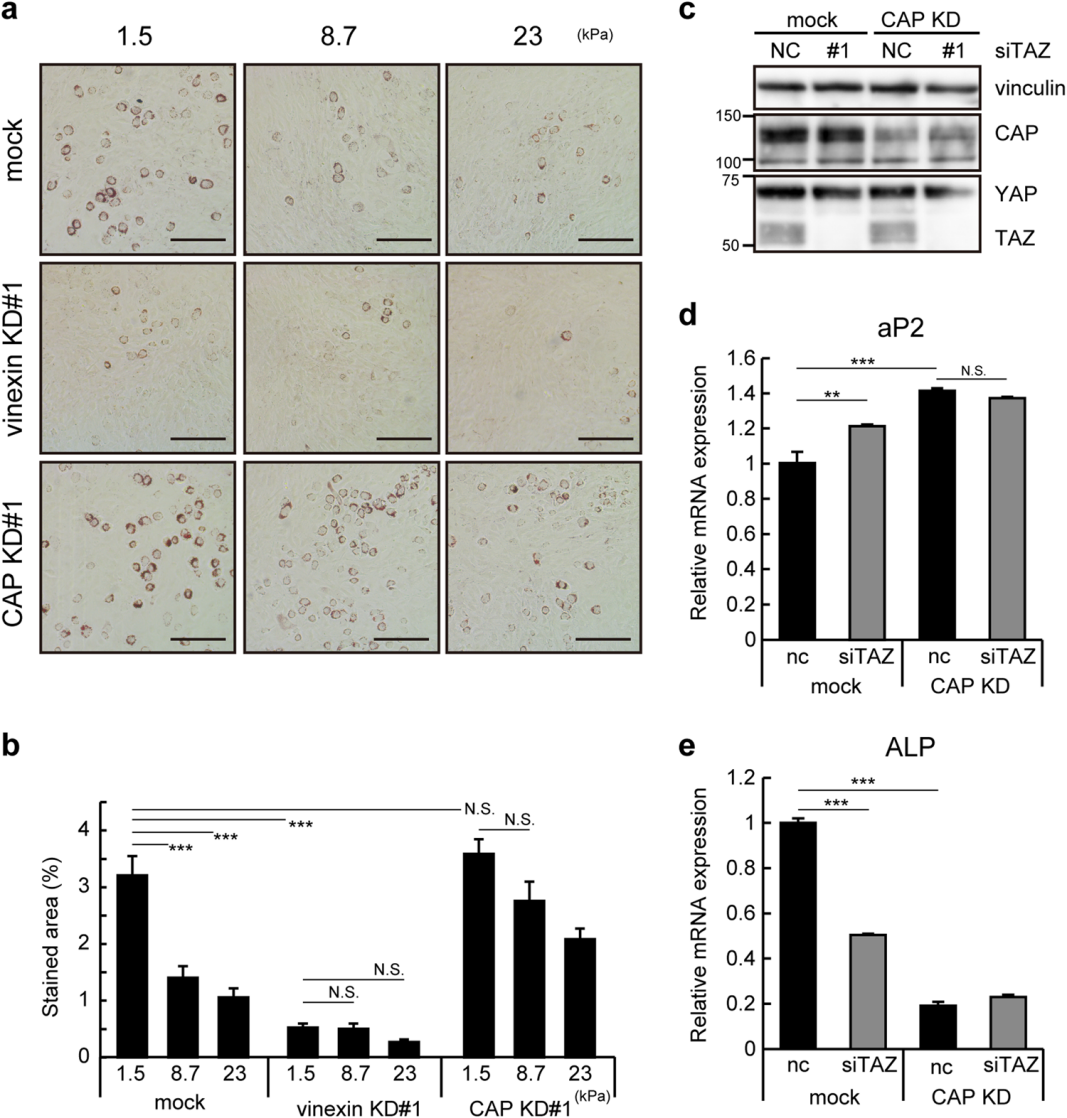
Vinexin and CAP regulated differentiation in an ECM stiffness-dependent manner. (**a**,**b**) Vinexin- or CAP-depleted cells were seeded onto PAA gels coated with collagen and induced to differentiate into adipocytes. Cells were stained with Oil Red O on day 6 after MDI treatment and representative images are shown. Scale bars: 200 μm. (**b**) Eight images were obtained from two independent experiments, and the percentage of the stained area was quantified using ImageJ. (**c**,**d**) CAP-depleted cells were transfected with negative control (siNC) or TAZ-targeted siRNA. (**c**) TAZ expression was analyzed with western blotting. Blots were cropped from full-size images (see Supplemental Information). (**d**) On day 6 after siRNA transfection, the expression of aP2 and ALP mRNA was quantified by qRT-PCR. The expression relative to control cells transfected with negative control siRNA from triplicate experiments is shown. Values represent the mean ± s.e.m. Each experiment was repeated twice. Statistical significance was determined by one-way ANOVA with Tukey’s test. **p < 0.01. ***p < 0.001.

Vinculin depletion attenuates the stiffness dependency of adipocyte differentiation in stiffness values ranging from 1.5 kPa to 8.7 kPa^17^. To test the effects of vinexin and CAP on ECM-stiffness dependent differentiation, vinexin- and CAP-depleted cells were cultured on PAA gels with stiffness values ranging from 1.5 kPa to 23 kPa. Consistent with a previous report, mock cells showed obvious ECM stiffness-dependent lipid droplet accumulation; rigid ECM (8.7 kPa and 23 kPa) significantly decreased the stained area to 43.8 ± 7.9% and 33.1 ± 6.0%, respectively, compared to those on soft ECM (1.5 kPa) (Fig. 6a,b). On the other hand, CAP depletion attenuated the ECM stiffness-dependent decrease in lipid droplet accumulation; rigid ECM decreased the stained area in CAP-depleted cells to only 76.9 ± 11.0% (8.7 kPa) and 58.1 ± 6.8% (23 kPa) of those on soft ECM. Interestingly, no significant difference was observed between control and CAP-depleted cells on soft ECM (1.5 kPa). In addition, vinexin depletion suppressed the accumulation of lipid droplets on PAA gels as well as on rigid (plastic) substrates, and abolished the ECM stiffness-dependency. These observations suggest that vinexin and CAP contribute to the ECM stiffness-dependent differentiation into adipocytes.

### TAZ mediates the effect of CAP on differentiation

Our previous study demonstrated that vinculin inhibits adipogenesis through TAZ^17^. To determine whether CAP suppresses adipocyte differentiation through YAP/TAZ, TAZ was depleted using siRNAs in control and CAP-depleted cells, then adipogenesis and osteogenesis were evaluated. The loss of TAZ expression was confirmed by western blotting (Fig. 6c). TAZ knockdown increased the expression of aP2 by 20.9 ± 7.6% and decreased the expression of ALP by 49.6 ± 1.2% in control cells, whereas TAZ knockdown did not affect aP2 and ALP expressions significantly in CAP-depleted cells (Fig. 6d). Similar results were obtained in YAP/TAZ double knockdown cells (Fig. S3). Taken together, these results suggest that CAP inhibits adipocyte differentiation by promoting TAZ nuclear localization.

## Discussion

Substantial effort has been made to understand the mechanism by which ECM stiffness regulates the differentiation of stem cells via adhesion molecules^6,33^. Our previous publication has revealed that SORBS family protein members, vinexin α and CAP, are required for inducing conformational changes of vinculin into a form that has high affinity for actin in MEFs when grown on rigid substrates^18^. Furthermore, vinculin is required for the ECM stiffness-dependent differentiation of MSCs. However, it is still unclear whether SORBS family proteins regulate mechanotransduction and differentiation of MSCs. Here, we show that both vinexin and CAP are required for the increase in CSK-resistant vinculin on rigid substrates and the promotion of the ECM stiffness-dependent YAP/TAZ nuclear localization. CAP is involved in the regulation of ECM stiffness-dependent MSC differentiation. In contrast, vinexin depletion inhibited the differentiation into adipocytes on substrates of any stiffness. Collectively, these observations indicate that CAP plays important roles in regulating the ECM stiffness-dependent differentiation of MSCs, whereas vinexin promotes adipocyte differentiation independent of ECM stiffness.

The most important finding in this study is that vinexin α and CAP regulate YAP/TAZ nuclear localization in an ECM stiffness-dependent manner. Vinexin α and CAP induce an ECM stiffness-dependent conformational change of vinculin^18^. In addition, vinculin regulates YAP/TAZ nuclear localization in a manner dependent on ECM stiffness. The P2 mutation of vinculin, which reduces interaction between vinculin with both vinexin α and CAP, attenuates this effect^17^. These observations suggest an important role for vinculin-vinexin α and vinculin-CAP interactions in the regulation of ECM stiffness-dependent YAP/TAZ nuclear localization in MSCs. Interestingly, depletion of vinexin or CAP clearly impaired the increase in nuclear localization of YAP/TAZ on substrates above 8.7 kPa stiffness; however depleted cells still exhibited a moderate increase in nuclear localization of YAP/TAZ on substrates of 8.7 kPa compared to that on 1.5 kPa. These results raises a possibility that vinculin-vinexin α and vinculin-CAP complexes function as mechanosensors for stiffness values ranging from 1.5 kPa to 42 kPa; however, it is likely that other mechanosensors also exist that are specific for a soft range of stiffness. One possible mechanosensor for a soft range of stiffness is talin. Talin is thought to be unfolded by a tensile force in order to expose VBS^10^. Previous investigations observed that increasing tension is applied to talin as the ECM stiffens within a soft range of stiffness; however, it remains constant at stiffness above 4 kPa^41,42^. An alternative explanation for the moderate response of YAP/TAZ localization is that the residual levels of vinculin-vinexin α and vinculin-CAP complexes in the depleted cells may be sufficient to promote the nuclear localization of YAP/TAZ on moderately rigid (8.7 kPa) substrates but not enough on rigid substrates.

Another important finding is that despite vinexin α and CAP depletion having a similar effect on the nuclear localization of YAP/TAZ, CAP suppresses differentiation of MSCs into adipocytes, whereas vinexin α promotes it. To the best of our knowledge, this study is the first report to demonstrate the opposite effect of vinexin α and CAP on the differentiation. Since TAZ binds to PPARγ, a master regulator for adipocyte differentiation, and suppresses its activity^43^, CAP-mediated suppression of adipocyte differentiation on rigid substrates can be explained by the regulation of YAP/TAZ nuclear localization. Indeed, TAZ knockdown in CAP depleted cells did not promote the adipocyte differentiation further. In contrast, the mechanism by which vinexin α promotes adipocyte differentiation is not clear. Both vinexin α and CAP bind various signaling molecules and regulate signaling pathways^24–26,44^. Thus, it is plausible that signals differently regulated by vinexin α and CAP contribute to the opposite effect. We examined activation of signaling molecules, including Akt, FAK, c-Src, AMPK, and JNK, in vinexin- and CAP-depleted cells but did not detect any significant differences (data not shown). Nuclear receptors may serve as possible pathways, since vinexin associates with several nuclear receptors, including estrogen receptor α and retinoic acid receptor γ^45,46^, and these receptors regulate adipocyte and osteoblast differentiation^47,48^. It is important to reveal signals that contribute to the opposite effect on differentiation in future research. The Hippo pathway is a well-known regulator of YAP/TAZ^49^. However, it is unlikely to mediate the effects of vinexin α and CAP because significant changes in the phosphorylation of YAP/TAZ or LATS, a kinase phosphorylating YAP/TAZ in the Hippo pathway, were not observed (data not shown). The FAs/actin cytoskeleton/nucleoskeleton axis may be a pathway involved in the regulation of the nuclear localization of YAP/TAZ^33,35–37,50^. As shown in our previous study, the actin-binding ability of vinculin is required for YAP/TAZ nuclear localization^17^. In addition, vinexin and CAP contribute to the aggregation of stress fibers at focal adhesions^22,51^, supporting this hypothesis. Finally, it is possible that vinexin and CAP regulate YAP/TAZ nuclear localization through kinases, such as the extracellular-regulated kinase (ERK). Vinexin and CAP directly bind to ERK^51–53^. In addition, Hwang et al. have reported that ERK regulates the nuclear localization of YAP/TAZ in hMSCs via a mechanism downstream of ECM stiffness^54^. Future examinations of these hypothesis will be interesting.

In the present study, we showed that the depletion of either vinexin or CAP reduced the conformational changes in vinculin and nuclear localization of YAP/TAZ on rigid substrates, suggesting that ‘both’ vinexin and CAP are required for these regulations. It is possible that vinexin and CAP work cooperatively to exert these effects. Indeed, one SORBS family protein, ArgBP2, is capable of binding to another ArgBP2 molecule through an association between its SH3 domain and a proline-rich cluster, leading to the formation of oligomers that stimulate interactions with its binding partners^55^. CAP variants can also form heterodimers among variants^38^. In addition, ArgBP2 and CAP form a heterodimer^55^. Alternatively, both vinexin and CAP may independently bind to different regions of vinculin to regulate its status. Two SH3 domains of vinexin or CAP bind to the second and third proline cluster in the proline-rich linker region of vinculin^21,22,56^, providing a possibility that one SH3 domain of each protein simultaneously binds to a different proline cluster in vinculin.

In contrast to ST2 cells, in which both vinexin and CAP are required, in triple SORBS protein KO/KD MEFs re-expressing one SORBS protein require ‘either’ vinexin or CAP for its ECM stiffness-dependent conformational change in vinculin^18^. This discrepancy may be ascribed to cell-type specific differences between MEFs and MSCs. Another possiblity is that the expression levels of SORBS proteins may affect the necessity of SORBS protein. SORBS proteins are re-expressed at higher levels in triple KO/KD MEFs than in wild-type MEFs^18^. The abundant expression might be sufficient for single SORBS proteins to exert their function. On the other hand, in the present study, the effects of vinexin and CAP were tested by depleting each protein and rescuing the effect by re-expression. Future studies should investigate these possibilities.

In summary, we demonstrate that vinexin and CAP, vinculin linker region-binding proteins, are involved in the ECM stiffness-dependent nuclear localization of YAP/TAZ. CAP is involved in ECM stiffness-dependent MSC differentiation, whereas vinexin predominantly promotes MSC differentiation into adipocytes on substrates of any stiffness. Together with the findings of our previous publication^17^, these observations suggest that the vinculin-CAP complex functions to regulate the ECM stiffness-dependent differentiation of MSCs into adipocytes by promoting the nuclear localization of YAP/TAZ. In contrast, vinexin regulates the differentiation via a mechanism independent of the YAP/TAZ pathway.

## Materials and Methods

### Plasmid construction

The small hairpin RNA for vinexin (#1 5′-GGTGAACGAACATTGGTATGA-3′, or #2 5′-CGGCTCAGGCTTTGTGATGATGG-3′) or CAP (#1 5′-GGACCTCCTCAATATAGATGA-3′, or #2 5′-GGAGACGTTGTTTACATCTAC-3′) were subcloned into pLKO.1-Puro vector from Open Biosystems (Huntsville, AL). Expression plasmids containing mouse vinexin α/β and CAP cDNA were described previously^4,18^. The cDNAs encoding vinexin and CAP resistant to shRNAs were generated by site-directed mutagenesis using In-Fusion® HD cloning kit (Clontech, Mountain View, CA) and subcloned into pCDH-EF1-IRES-hygro vector from System Biosciences (Mountain View, CA). Generation of monomeric GFP-tagged vinculin T12 mutant were described previously^16^.

### Antibodies and Reagents

Mouse anti-vinculin (V9131, dilution ratio: 1/20,000 (WB), 1/500 (IF)), rabbit anti-CAP (SORBS1) (HPA027559, 1/2,000 (WB), 1/100 (IF)) and anti-β tubulin (T4026, 1/2,000) antibodies were purchased from Sigma (Saint Louis, MO). Mouse anti-β-actin antibody (ab6276, 1/10,000) was purchased from Abcam (Cambridge, UK). Mouse anti-YAP (sc-101199, 1/100 (IF), 1/1000 (WB)), and rabbit anti-ERK2 (sc-154, 1/10,000) were purchased from Santa Cruz Biotechnology (Santa Cruz, CA). Rabbit anti-aP2 (#3544, 1/2,000) antibodies was purchased from Cell Signaling Technology (Boston, MA). Rabbit anti-vinexin and anti-ArgBP2 polyclonal antibodies were described previously^18,22^. Alexa Fluor 568 phalloidin and 633 phalloidin was purchased from Thermo Fisher Scientific (Rockford, IL). Type I collagen was purchased from Nitta Gelatin (Osaka, Japan). Insulin and 3-Isobutyl-1-methylxanthine (IBMX) were purchased from Sigma.

### Cell culture and differentiation

ST2 cells, a mouse bone-marrow derived mesenchymal stem cell line, were obtained from RIKEN BRC (Tsukuba, Japan). ST2 cells were maintained in RPMI1640 media (Sigma) supplemented with 10% fetal bovine serum (FBS; Sigma) at 37 °C in a humidified atmosphere containing 5% CO_2_. PAA gel substrates were prepared as described^4,17^. The elastic moduli of the gels ranged from 1.5 kPa to 42 kPa. Adipocyte differentiation was induced as described previously^17^. Osteoblast differentiation was induced by culturing cells with αMEM (Sigma) containing 50 μg/ml ascorbic acid, and the medium was replaced every 2 days.

### Establishment of vinexin- and CAP-depleted cells and re-expressing cells

Vinexin- and CAP-depleted cells and vinexin α or CAP re-expressing cells were established using lentiviruses as previously described^4,17^. Briefly, lentiviruses were generated by transfecting pMD2.G, psPAX2, pRSV-Rev, pMDLg/pRRE (Addgene, Cambridge, MA), and pLKO.1 vectors (for knockdown) or pCDH vectors (for expression) into HEK293T cells using Lipofectamine® LTX and PLUS™ Reagent (Thermo Fisher Scientific). Cells were infected with lentiviruses, followed by incubating with medium containing 1 μg/ml puromycin or 100 μg/ml hygromycin B.

### siRNA-mediated knockdown

ST2 cells were transfected with 2 nM StealthTM RNAi siRNAs against TAZ (siTAZ#1 5′-GGAAGGUGAUGAAUCAGCCUCUUGG-3′, siTAZ#2; 5′-GGAGUCCUUCUUUAAGGAGCCCGAU-3′; Invitrogen, Carlsbad, CA), YAP (5′-CCAAGACAUCUUCUGGUCAAAGAUA-3′; Invitrogen) or control siRNA (Stealth™ RNAi Negative Control Medium GC Duplex#3, Cat. No. 12935-113) using Lipofectamine® RNAiMAX Reagent (Invitrogen). Lipofectamine complexes were diluted with 500 μl of OPTI-MEM (Gibco) and transfected into 1.0 × 10^5^ cells in 6 well plates. The medium was changed twenty-four hours after transfection.

### Oil Red O staining, ALP staining, Alizarin Red S staining and quantification

Oil Red O staining was performed as described^17^. ALP activity staining was performed using the TRACP & ALP double-stain kit (TaKaRa, Ohtsu, Japan). For Alizarin Red S staining, cells were fixed with 10% formalin solution for 15 min. After washing with distilled water, cells were stained with 1% Alizarin Red S solution (Sigma) (pH 6.37 in ammonia solution) for 30 min. After washing with distilled water, images of stained cells were obtained by a microscope (Nikon ECLIPSE TE300-2) equipped with Leica MC120. For Oil Red O staining and Alizarin Red S staining, the stained area per total area were quantified using ImageJ software. For ALP staining, the staining intensity in each image was quantified.

### Quantitative real-time PCR (qRT-PCR)

qRT-PCR was performed as previously described^17^. Briefly, total RNA was extracted using RNeasy mini Kit (QIAGEN, Hilden, Germany), and cDNAs were synthesized using Super Script reverse transcriptase III (Invitrogen). qRT-PCR analysis was carried out with Step One^TM^ Real-Time PCR Systems (Applied Biosystems) using THUNDERBIRD® SYBR qPCR Mix (TOYOBO, Osaka, Japan). Relative expression levels to internal control 36B4 are presented. Sequences of specific primers used in this paper are described in s previous study^17^.

### Immunostaining and quantification of FAs and YAP/TAZ nuclear localization

Immunostaining and quantification were performed as described previously^4,17^. Briefly, to analyze total vinculin, cells were fixed with 1.5% paraformaldehyde at RT for 45 min, followed by permeabilization with PBS containing 0.2% Triton X-100 for 5 min at room temperature. To analyze CSK-resistant vinculin, cells were first treated twice with CSK buffer (0.1% Triton X-100, 10 mM PIPES, pH 6.8, 50 mM NaCl, 3 mM MgCl_2_, and 300 mM sucrose) at 4 °C for 30 sec, followed by fixation with 4% paraformaldehyde at room temperature. Images were obtained with LSM700 confocal microscope (Carl Zeiss, Oberkochen, Germany) equipped with a ×40 Plan-APOCHROM objective lens. To obtain quantified data from single cells, neighboring cells in the images were erased. Quantification of the total number and average area of vinculin at FAs per cell was performed using ImageJ. Focal adhesions were classed as structures of 1–20 µm^2^ in the ‘Analyze Particle’ command. To analyze YAP/TAZ nuclear localization, cells were fixed with 4% paraformaldehyde and permeabilized with 0.2% Triton X-100 for 5 min at room temperature. Images were obtained with LSM700 confocal microscope with a ×40 Plan-APOCHROM objective lens, or with a Nikon C2 confocal microscope (Nikon, Tokyo, Japan) equipped with a ×40 Plan-APO λ objective lens. The intensity of the same areas in nucleus and cytosol (just outside the nucleus) were quantified using ImageJ. The nuclear regions were defined by Hoechst or TOTO-3 staining. Then, the nucleus-to-cytosol intensity ratio was calculated.

### Statistical analysis

Statistical analyses were performed using Origin 2017 software. Values according to the normal distribution were analyzed with Tukey’s honest significant difference test after one-way ANOVA and Student’s t-test for comparison among three or more groups and between two groups, respectively. Values that were not normally distributed were analyzed with Mann-Whitney U-test after Kruskal-Wallis ANOVA for comparisons among three or more groups.

### Summary statement

Vinexin α and CAP, two of SORBS proteins, regulate YAP/TAZ nuclear localization. CAP plays a crucial role in ECM stiffness-dependent differentiation of mesenchymal stem cells.

## Electronic supplementary material

Supplementary Information
