# Supplementary material for: Vinexin family (SORBS) proteins regulate mechanotransduction in mesenchymal stem cells
Source: Sci Rep. 2018 Aug 1;8:11581. doi: 10.1038/s41598-018-29700-3 (PMC6070524; doi:10.1038/s41598-018-29700-3)
Supplement: Supplementary file 1 — Supplementary Information [file 41598_2018_29700_MOESM1_ESM.pdf]

# Supplementary information

## Vinexin family (SORBS) proteins regulate mechanotransduction in mesenchymal stem cells

Mito Kuroda[1], Kazumitsu Ueda[1,2], and Noriyuki Kioka

1Division of Applied Life Sciences, Graduate School of Agriculture, Kyoto University, Sakyo, Kyoto 606-8502, Japan,
2Institute for Integrated Cell-Material Sciences (iCeMS), Kyoto University, Sakyo, Kyoto 606-8507, Japan

+Address correspondence to Noriyuki Kioka, Ph.D.,

**Fig.S1 Expression of SORBS family proteins in ST2 mesenchymal stem cells**

(A) Schematic of the domain structure of SORBS proteins (vinexin $\alpha$ and $\beta$, and CAP). (B, E, F) Cell lysates were analyzed using western blotting. $\beta$-tubulin and ERK2 were used as loading controls. A black arrowhead indicates endogenously expressed vinexin $\alpha$. (C, D) The localization of vinculin, vinexin, and CAP were analyzed with immunostaining.

**Fig.S2 (Related to Fig. 1 and Fig. 4)**

(A) Vinexin- and CAP-depleted cells were stained with Cell Mask Orange. (B) Cell area and aspect ratio were quantified using ImageJ. (C) Equal amounts of cell lysates were analyzed by western blotting using the anti-vinculin, anti-vinexin, anti-CAP, and anti-PPAR$\gamma$ antibodies.

**Fig.S3 (Related to Fig. 6)**

(A, B) Experiments were performed as described in Fig.6 using a mixture of 0.5 nM YAP siRNA and 1.5 nM TAZ siRNA.

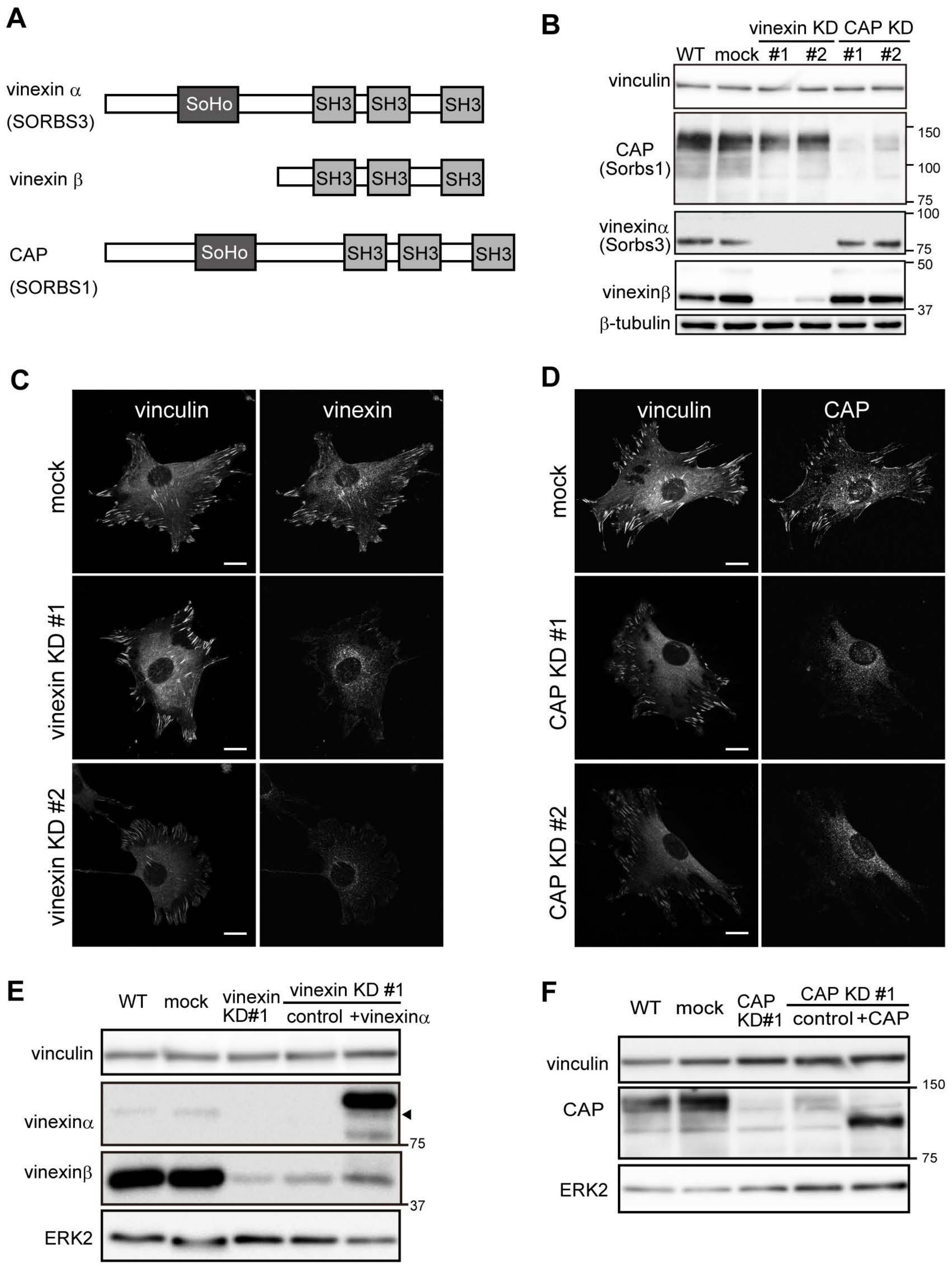


Fig.S1 Kuroda et al.

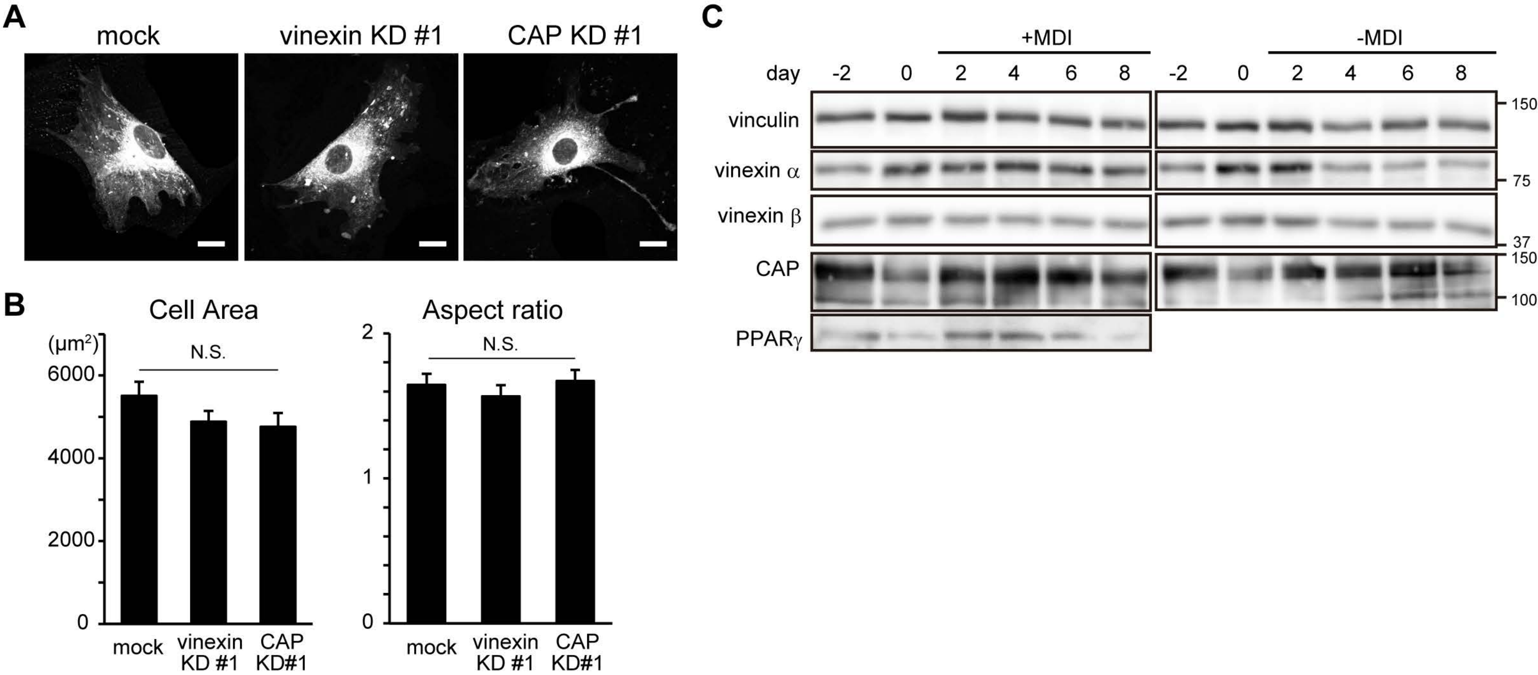


Fig.S2 Kuroda et al.

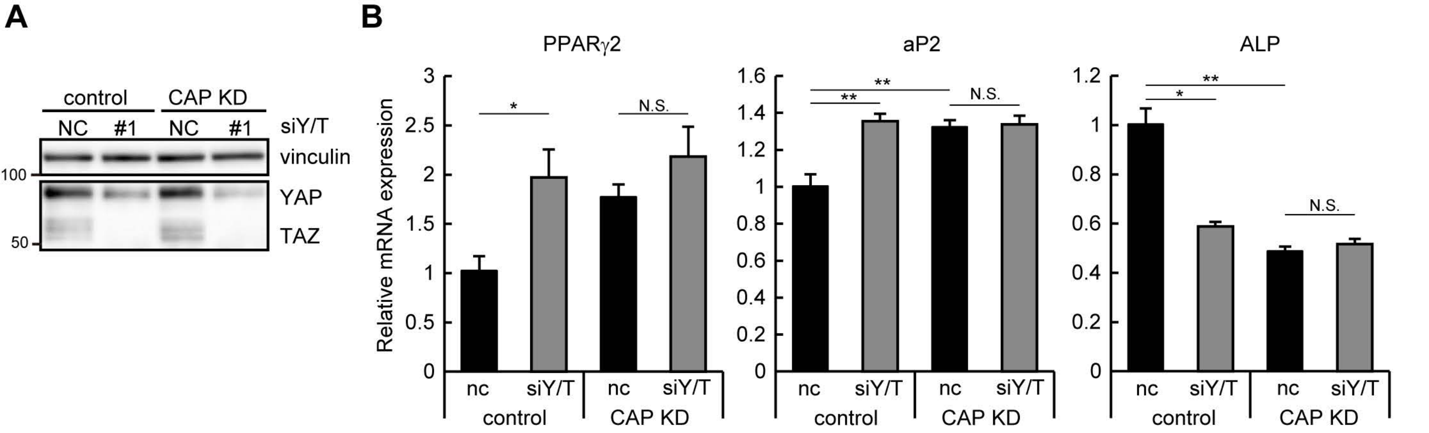


Fig.S3 Kuroda et al.

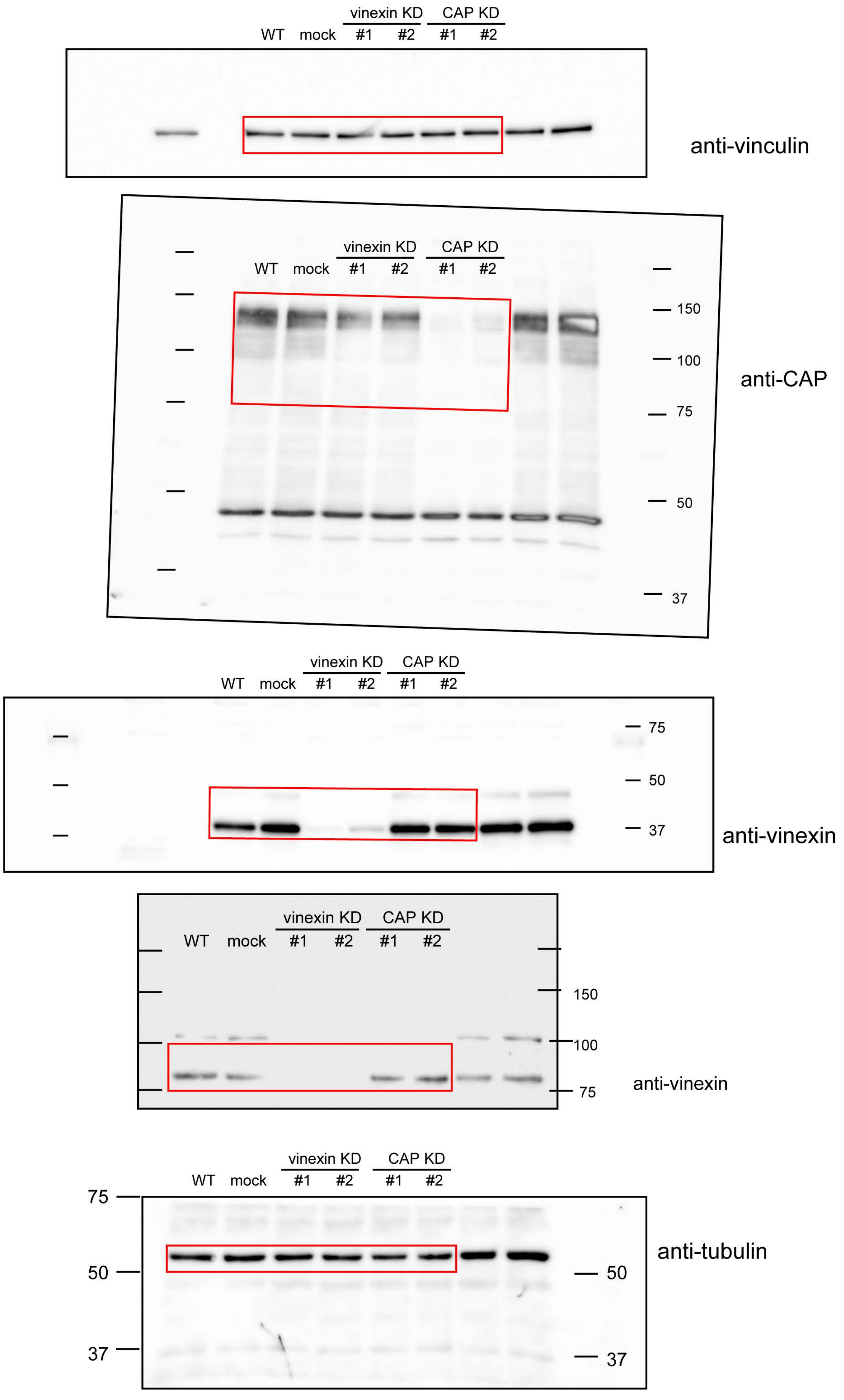


Figure S1B western

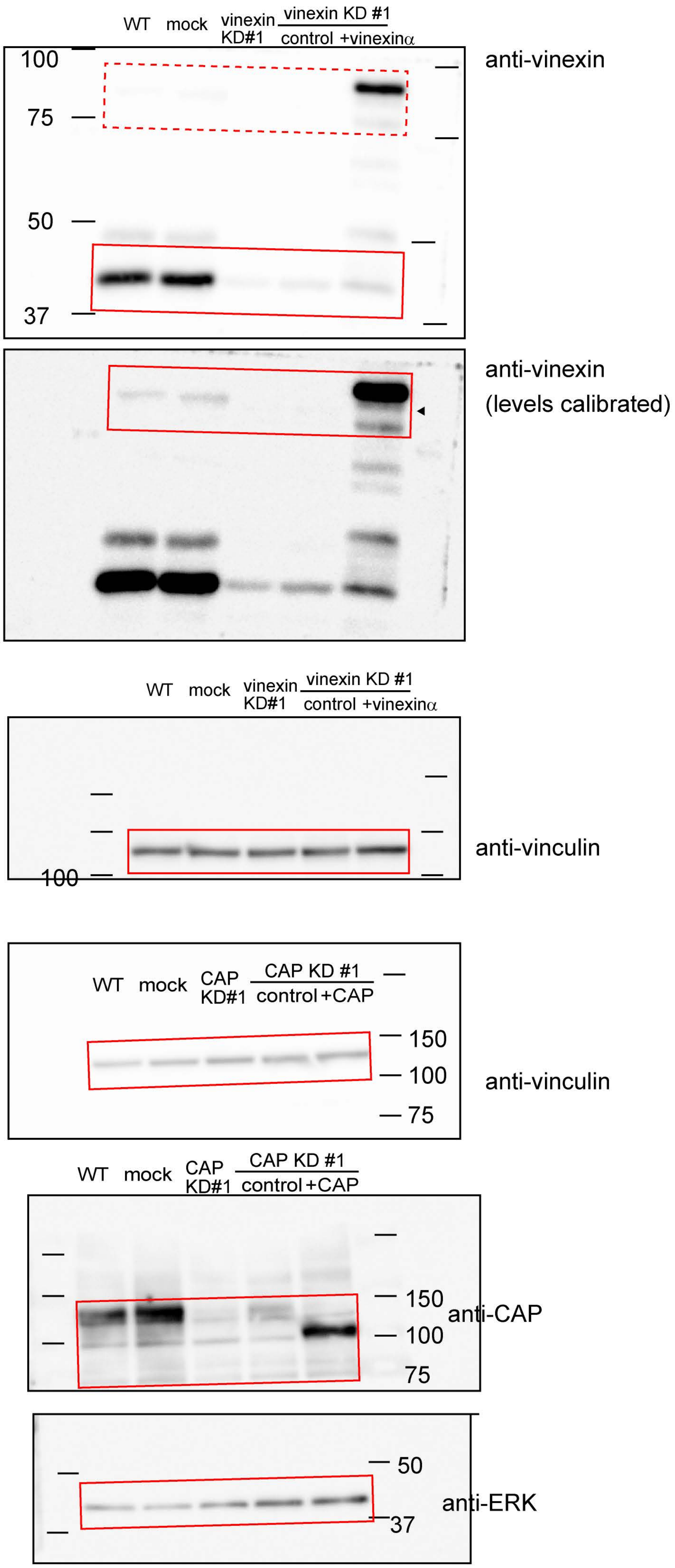


Figure S1E,F western

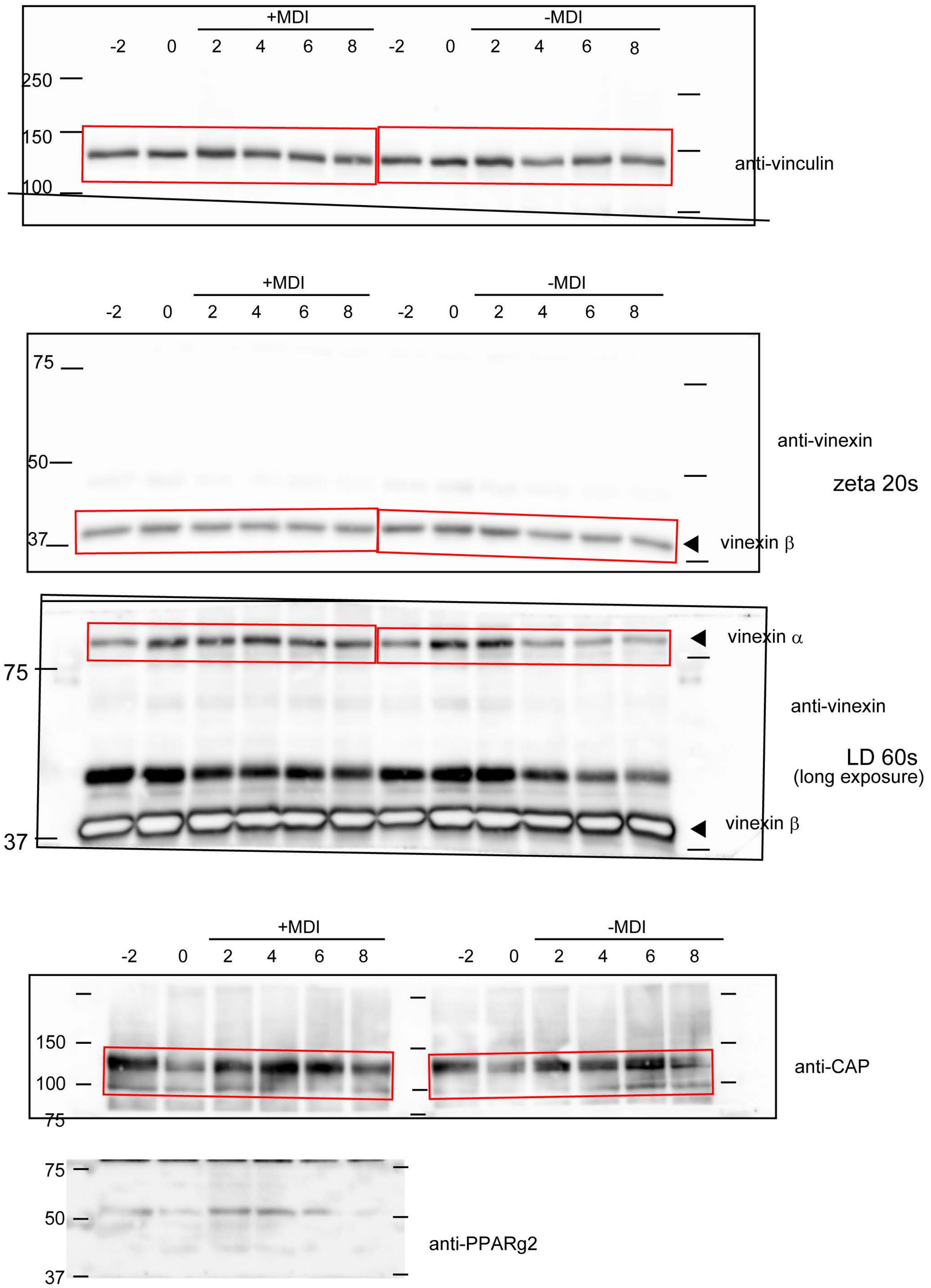


Fig S2C western

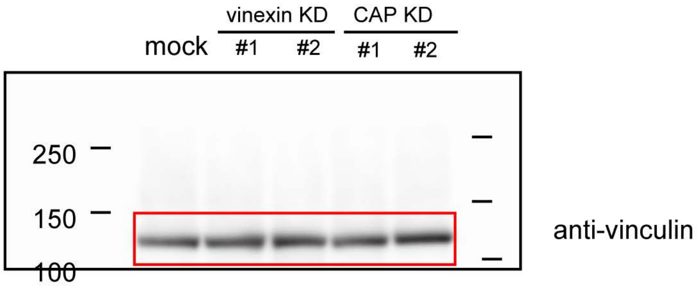


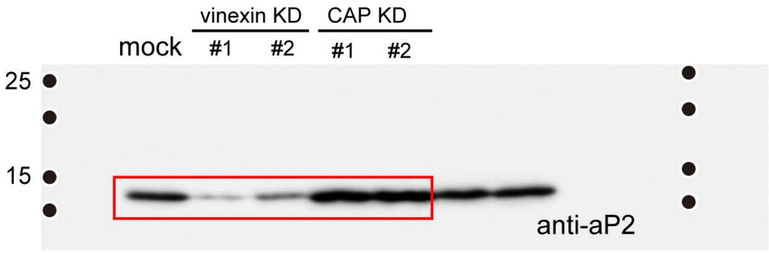


Fig. 4B western blot data

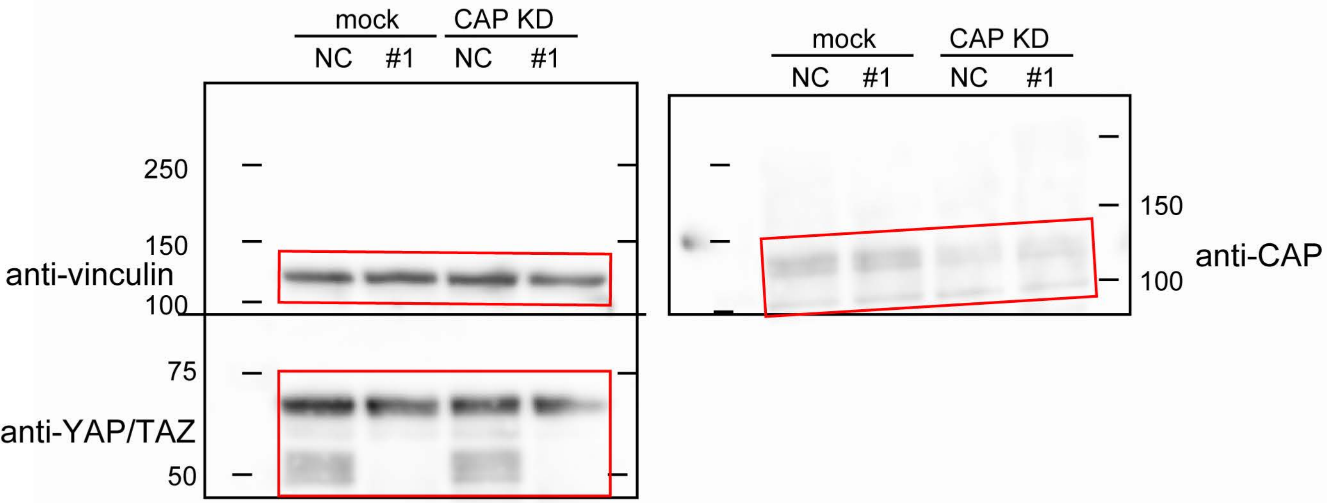


Fig. 6C western blot data

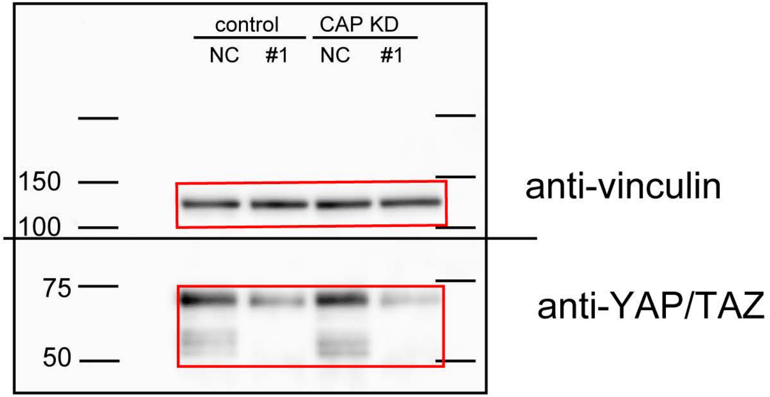


Figure S3 western
